# Interference between items stored for distinct tasks in visual working memory

**DOI:** 10.3758/s13414-023-02657-w

**Published:** 2023-01-31

**Authors:** Stefan Czoschke, Benjamin Peters, Jochen Kaiser, Christoph Bledowski

**Affiliations:** 1 Institute of Medical Psychology, Goethe University, Heinrich-Hoffmann-Strasse 10, D-60528 Frankfurt am Main, Germany; 2 Mortimer B. Zuckerman Mind Brain Behavior Institute, Columbia University, New York, NY USA

**Keywords:** Visual working memory, Perception and action

## Abstract

The action perspective on working memory suggests that memory representations are coded according to their specific temporal and behavioral task demands. This stands in contrast to theories that assume representations are stored in a task-agnostic format within a “common workspace”. Here, we tested whether visual items that are memorized for different tasks are stored separately from one another or show evidence of inter-item interference during concurrent maintenance, indicating a common storage. In two experiments, we combined a framing memory task (memorize a motion direction for continuous direction report) with an embedded memory task (memorize a motion direction for a binary direction discrimination) that was placed within the retention period of the framing task. Even though the temporal and action demands were item specific, we observed two types of interference effects between the items: The embedded motion direction was (1) repulsed away and (2) degraded in precision by the motion direction of the item in the framing task. Repulsion and precision degradation increased with item similarity when both items were concurrently held in working memory. In contrast, perceptual and iconic memory control conditions revealed weaker repulsion overall and no interference effect on precision during the stimulus processing stages prior to working memory consolidation. Thus, additional inter-item interference arose uniquely within working memory. Together, our results present evidence that items that are stored for distinct tasks to be performed at distinct points in time, reside in a common workspace in working memory.

Working memory is crucial for goal-oriented behavior and adaptive functioning as it enables us to bridge the time periods between information uptake and behavioral utilization. Working memory content, thus, plays a pivotal role in actively guiding upcoming actions. However, as complex behavior often consists of interposed or nested tasks, concurrently maintained information sometimes serves distinct behavioral purposes and belongs to independent task sets. While short-term storage of items for a single task is well understood, a rarely addressed question is to what extent the principles of memory retention are governed by the prospective action plans associated with individual items.

Working memory is a capacity-limited store that can maintain only a few pieces of information with high fidelity. Within working memory, items are not stored in isolation but engage in mutual interactions that affect the individual item representations. For example, inter-item competition can lead both to decreases in *precision* (i.e., a wider distribution of responses around the true value across trials; Bouchacourt & Buschman, 2019; Jiang et al., 2016; Oberauer & Lin, 2017; Pertzov et al., 2017), and to systematic *biases* (i.e., shifts of the mean of the response distribution away from the true value). These biases manifest themselves either as repulsion, where items are reported to be more dissimilar to other items maintained in working memory (Czoschke et al., 2019; Kang & Choi, 2015; Scotti et al., 2021), or as attraction, where items are reported to be more similar to other items than they actually are (Czoschke et al., 2020; Huang & Sekuler, 2010; Saito et al., 2020; Wildegger et al., 2015). Such repulsion and attraction biases suggest that stored materials reside in a common frame that relates item representations to each other prior to being accessed for action. Crucially, in most working memory studies, items are stored as task-equivalent candidates, with each item potentially becoming the response target for report or recognition at the end of the trial. Those studies present convincing evidence that material that is concurrently stored for the same purpose resides in a competitive relationship. A simple model to account for these findings would be to think of working memory as a store that integrates and stores all information that is relevant for future actions in a common workspace (e.g., Compte et al., 2000; Franconeri et al., 2013; Johnson et al., 2008; Lin & Luck, 2009; Oberauer & Lin, 2017; Schneegans & Bays, 2017; Swan & Wyble, 2014).

Recent theoretical proposals, however, have emphasized that working memory serves not only the collection and conservation of past impressions but that it provides task-optimized information for upcoming actions (Nobre & Stokes, 2019). The storage characteristics of an item in working memory might thus depend on its specific behavioral purpose (van Ede, 2020)—that is, memory items might be organized and separated by the “when” and “how” of their intended use rather than residing in a common, task-agnostic format until they are needed for recall (Myers et al., 2017; van Ede et al., 2020). From this line of reasoning, the mnemonic code that represents an item would depend entirely, or as an additional layer of representation, on the type of task it is stored for, and on whether it is relevant for the immediate task at hand or needs to be retrieved later for a subsequent action.

Evidence for different memory formats depending on the expected time-point or *temporal order of use* came from behavioral as well as neuroimaging studies (Olivers & Roelfsema, 2020). These studies have repeatedly shown that a memory item that is expected to be required imminently for the upcoming task is maintained in a qualitatively different state of accessibility than an item that is stored for later use (Christophel et al., 2018; de Vries et al., 2020; LaRocque et al., 2017; Lewis-Peacock et al., 2012; Peters et al., 2018). In particular, the currently prioritized item allows a speeded response (McElree, 2001), guides attention towards task-relevant sensory input (de Vries et al., 2020), and seems to employ different maintenance mechanisms and/or brain regions than nonprioritized items (Christophel et al., 2018; de Vries et al., 2020; LaRocque et al., 2017; Lewis-Peacock et al., 2012).

Apart from the temporal order of use, the format of a memory representation might depend also on the *type of action* it is stored for (e.g., reproduction or recognition; Nobre & Stokes, 2019). Goal-specific stimulus processing occurs already at the stages of encoding and maintenance by processing primarily those features that are relevant for the intended action (Allport, 1987; Heuer et al., 2020; Monaco et al., 2018). Additionally, memory representations have been speculated to get recoded from a perception-conserving, sensory representation into a task-adapted format that suits the specific response demands of the task (Myers et al., 2017). Accordingly, neural signatures of memory retention have been shown to depend on the type of recall task that the participants expect (Muhle-Karbe et al., 2017; Warden & Miller, 2010).

The neural and functional characteristics that individualize memory representations according to their unique temporal and behavioral task demands have been argued to serve several adaptive functions, such as increasing behavioral readiness (Boettcher et al., 2021; Myers et al., 2017), reducing interference between ongoing perception and memory content (de Vries et al., 2020) and avoiding cross-item interference between current and subsequently relevant memory items (Myers et al., 2017; Nobre & Stokes, 2019). This action perspective on working memory thus suggests that memory representations are coded and segregated according to their specific temporal and behavioral task demands rather than residing in a task-agnostic “common workspace” until recall.

Task-agnostic and task-specific memory representations are of course not necessarily mutually exclusive alternatives. As several sites of the brain have been identified to carry memory-related information about the same object (e.g., Christophel et al., 2017; Czoschke et al., 2021; Yan et al., 2021), multiple representational formats might coexist and enrich each other when dealing with changing demands of our environment.

Here, we tested for a strong separation account of task-related memory coding. Namely, whether visual working memory items that are stored for distinct actions, which were due at distinct points in time, are maintained as isolated representations or show evidence of inter-item interference during a concurrent maintenance period. To this end, we asked participants to memorize two motion directions sequentially within a trial. The first motion direction was memorized for a continuous direction report task that was probed at the end of the trial (framing task). During the retention interval of the first item we asked subjects to memorize a second motion direction for a shortly delayed binary decision in a direction discrimination task unrelated to the first item (embedded task). Thus, the framing task and the embedded task differed with respect to both, the expected type of task (continuous direction report versus binary direction discrimination) and the temporal order (the embedded item was always probed prior to the framing item). This trial design created ideal circumstances for separating both items by task and timing demands during a concurrent retention period. If both items were indeed stored as isolated representations, we would expect no interactions between them on the memory level. In contrast, if both items were stored in a common workspace, we would expect systematic interactions between them with regard to precision and systematic biases. To be able to observe inter-item specific interactions, we manipulated the similarity of the motion directions, since both the precision and the magnitude of biases have been shown to be modulated by inter-item similarity (Czoschke et al., 2019; Jiang et al., 2016; Oberauer & Lin, 2017). Moreover, to determine whether the observed effects were indeed due to interactions between consolidated representations within working memory, we compared precision and biases of the embedded item in the memory condition with control conditions in which the response probe appeared either during the presentation of the embedded item (perception condition in Experiment 1), or directly after the offset of the embedded item (iconic memory condition in Experiment 2).

Importantly, our experiment was designed to focus specifically on proactive effects (i.e., effects measured by the embedded direction discrimination task). Data observed in the framing continuous recall task can be affected by processes that occurred during or after the execution of the embedded task, like, for example, retrieval-induced repulsion of the framing item (Kang & Choi, 2015), removal of the embedded item from memory (Lewis-Peacock et al., 2018), or transition of the framing item from an active to a passive memory state (Peters et al., 2018). Hence, any effects on precision and systematic biases of the framing item could not be unequivocally related to its interaction with the embedded item before the embedded task is executed.

## Experiment 1

The first experiment contrasted the representational shift and precision change that the framing item induced onto the embedded item during concurrent maintenance in working memory with a control condition that measured the effect of the framing task onto the mere perceptual appearance of the embedded item. Working memory content, maintained for later use, has been shown to affect the ongoing perceptual processing stream. For example, working memory content has been shown to alter the appearance of subsequently encoded visual stimuli already on the perceptual level by repelling (i.e., increasing the perceived dissimilarity between the items; Kang et al., 2011; Scocchia et al., 2013) or attracting (i.e., increasing the perceived similarity between the items; Teng & Kravitz, 2019) stimulus perception relative to the concurrently held memorandum. Our experimental design allowed us to distinguish inter-item interactions between consolidated memory representations from effects occurring during perceptual processing of the embedded item. If both items are maintained strictly separated in working memory, we would expect no inter-item interference beyond what might be induced during perceptual processing. In contrast, additional interference in the working memory condition would suggest a joint storage principle as in a “common workspace.”

### Methods

#### Participants

Twenty-four adults (17 females; age 20–33 years; *M* = 23.68, *SD* = 3.36) participated in the experiment after giving written informed consent. The study was approved by the local ethics committee. All participants reported normal or corrected-to-normal visual acuity. They were naïve to the purpose of the experiment and were either paid (€10/hr) or received course credit for their participation. Five subjects were excluded from data analysis due to failure of the curve-fitting procedure to estimate psychometric functions. Upon visual inspection, data of three participants were consistent with a complete guessing response strategy, one participant showed a reverse response pattern consistent with a mix-up of the response buttons, and one subject had an outlier width of the psychometric function of >2.5 standard deviations of mean. One subject dropped out after the first session. This left data from the remaining 18 participants (14 females; age 20–33 years; *M* = 24.06, *SD* = 3.65) for analysis.

The sample size was based on previous studies from our laboratory (Czoschke et al., 2019, 2020) that showed reliable proactive item interactions for sample sizes of about 16 participants. To ensure sufficient data sets after data-based exclusion and drop-out between sessions, we invited 24 participants to the experiment.

#### Stimuli and apparatus

Random dot patterns (RDP) were presented at the center of the screen of an LCD monitor (refresh rate 60 Hz). Participants viewed them from a distance of 70 cm in a dimly lit room. RDPs consisted of 200 white dots on a black background, with each dot covering approx. 0.11° of visual angle. All dots were displayed within an invisible circular aperture of approx. 10.74° of visual angle in diameter and moved at 2.5° per second with 100% coherence. The dots were placed randomly within the circular aperture at stimulus onset and repositioned on each frame by 1.93 pixels in the direction of motion. Dots reaching the edge of the circular aperture were repositioned randomly on the edge of the opposing semicircle, thus keeping dot density constant throughout the presentation. Each trial contained two RDPs. The motion direction of the first RDP was randomly drawn from a set of 36 possible directions ranging from 0° to 350° in steps of 10°. The motion direction of the second RDP could deviate by ±30°, ±60°, or ±90° from the first stimulus in a balanced fashion. MATLAB R2010a and the Psychophysics Toolbox (Brainard, 1997) were used to generate and display the stimuli.

#### Procedure

**Fig. 1:**
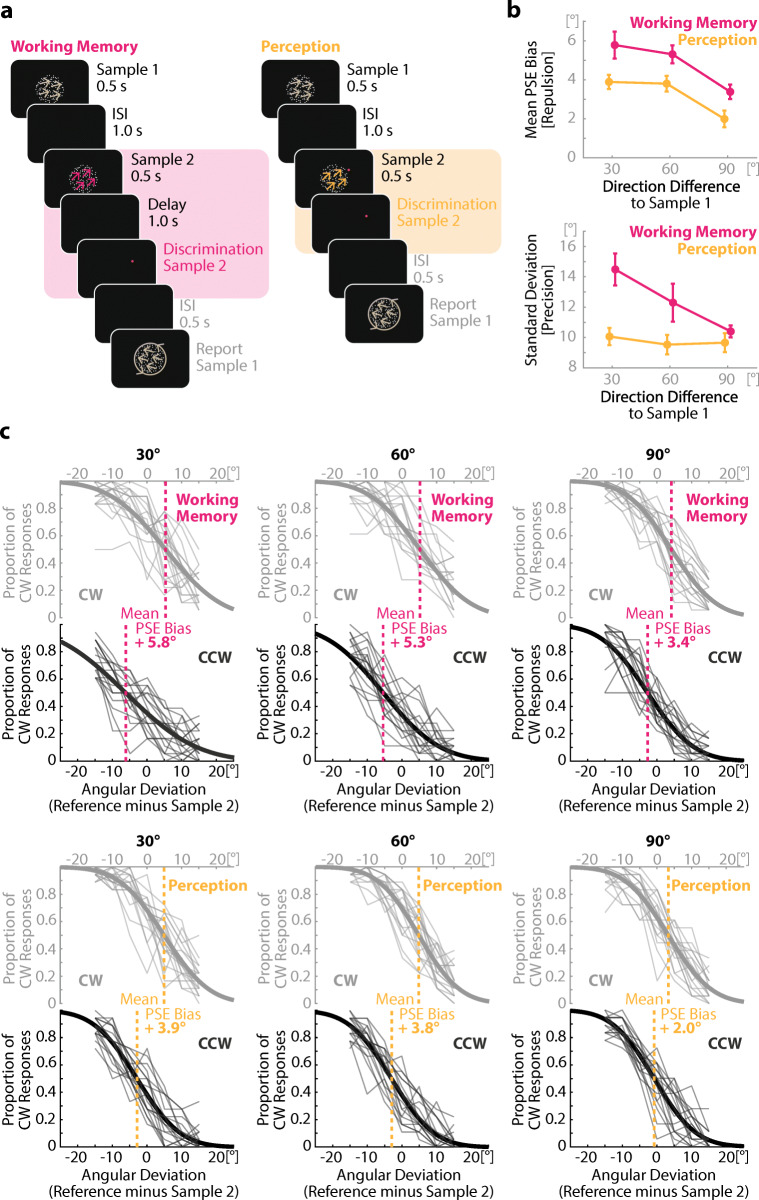
Design and results of Experiment 1. **a** Schematic depiction of an experimental trial of Experiment 1 in the working memory (left) and perceptual (right) condition. In both conditions, subjects first memorized one motion direction (arrows for illustration only) of a random-dot-pattern (RDP) for a continuous report at the end of the trial (framing task). In the embedded task, a second motion direction either had to be memorized for a one second delayed binary discrimination task (working memory condition) or be discriminated directly upon stimulus onset (perception condition). Note that the colors in the trial depiction are for illustrative purposes only. Both RDPs were equally white in the experiment. **b** Repulsive bias (top) and precision (bottom) of Sample 2 in the embedded task for different levels of similarity (30°, 60°, 90°) and different conditions (working memory, perception). Sample 2 in the embedded task was repulsed by Sample 1 of the framing task in both conditions, as indicated by a shift of the PSE away from the motion direction. This repulsion effect was stronger when Sample 2 was similar to Sample 1 and stored in working memory as compared with when it was dissimilar to Sample 1 and only processed in perception. We also found lower precision (higher *SD*) for Sample 2 in the working memory compared with the perception condition. This effect, however, was strongly modulated by the motion direction similarity between both samples, indicating that the precision decrease was not due to a general accumulation of noise during the prolonged delay period, but the result of competition between both memory items during concurrent maintenance. Error bars represent within-subject standard errors (Morey, 2008). **c** Detailed visualization of the repulsive bias including individual subjects’ data. Mean PSE bias was calculated as half the distance between the PSE of trials in which Sample 2 was oriented clockwise relative to Sample 1 (CW) and the PSE of trials in which Sample 2 was oriented counterclockwise relative to Sample 1 (CCW). The figures in **c** show the mean performance on the direction discrimination task at the level of individual subjects (thin lines). Superimposed is the mean psychometric function (thick line). The dashed line shows the point of subjective equality (PSE) per condition. For CW trials, a shift of the psychometric function to the right indicates repulsion of Sample 2 from Sample 1; for CCW trials, a shift to the left indicates repulsion. (Color figure online)

Figure 1a depicts the trial structure. Each trial consisted of two tasks: a framing working memory continuous direction report task and an embedded binary direction discrimination task. Each trial began with a 1-s fixation period. The two stimuli then appeared sequentially for 0.5 s each with an interstimulus interval of 1 s. Participants memorized the motion direction of the first stimulus for a continuous report task at the end of the trial. The structure of the embedded task depended on the condition: In the perceptual condition the second stimulus appeared together with a reference point (a red dot), placed at the outer edge of the RDP. The participants were asked to indicate via button press whether the motion direction of the second stimulus was clockwise or counterclockwise to the reference point. The reference point remained visible after the offset of the RDP until response. In the working memory condition, the reference point appeared 1 s after offset of the second stimulus and remained on screen up to the response. The reference point could deviate by ±15°, ±10°, ±5° or 0° from the direction of the second stimulus. 0.5 s after the binary response, a randomly oriented clock hand appeared in the center of the screen. Participants adjusted the clock hand via horizontal movements of a computer mouse to match the direction of the first stimulus. If the adjusted direction differed by more than 30° from the true direction, the clock hand switched to the true direction for 0.5 s as feedback. Throughout a trial, a 0.11° white square located at the center of the screen served as a fixation point. Subjects were instructed to fixate the square for the duration of the experiment while performing the task. The experiment consisted of 1,512 experimental trials, 756 trials per condition (perception, working memory) comprising 252 repetitions per inter-item similarity step (30°, 60°, 90°) with 36 repetitions per reference probe deviation each. Participants performed 30 practice trials at the beginning of each session that were excluded from data analysis. Conditions (perception, working memory) were presented in a block design with one condition per session, counterbalanced across participants. The experiment lasted about 2 hours per session and comprised two sessions on different days.

#### Data analysis

The experiment was designed to focus on the analysis of the behavioral data from the embedded direction discrimination task. We calculated the psychometric functions for each condition (perception, working memory × inter-item similarity) by fitting a cumulative gaussian to the direction discrimination, using the EzyFit toolbox for MATLAB (Moisy, 2011). From the resultant psychometric functions, we extracted the point of subjective equality (PSE) as a location parameter that indicates the shift of the representation along the feature space—that is, its systematic bias and the standard deviation (*SD*) of the distribution to indicate the precision of the representation. To analyze biases of the embedded item relative to the direction of the framing stimulus, we performed this procedure separately for trials in which the direction of Sample 2 was oriented clockwise relative to Sample 1 (CW trials) and for trials in which Sample 2 was oriented counterclockwise relative to Sample 1 (CCW trials). Biases were then calculated as half the distance between the PSE of trials in which Sample 2 was oriented clockwise relative to Sample 1 (CW) and the PSE of trials in which Sample 2 was oriented counterclockwise relative to Sample 1 ( (PSE_cw_ – PSE_ccw_) / 2 ). With a positive sign indicating repulsion (i.e., the representation of the embedded stimulus was shifted away from the first stimulus) and a negative sign indicating attraction (i.e., the representation of the embedded stimulus was shifted towards the first stimulus). Precision was calculated as the mean *SD* of the psychometric functions of CW and CCW trials. Inferential statistics were based on a 2 × 3 factorial design with the factors processing level (perceptual vs. working memory) and inter-item similarity (30°, 60°, 90°). We compared the effect of the framing memory item on the PSE shift and the *SD* (i.e., the representational shift and the fidelity) of the psychometric function of the embedded item. All reported analyses of variance (ANOVAs) are based on a repeated-measures design. An alpha level of .05 (two-tailed) was set for all statistical tests.

### Results

Experiment 1 tested whether the item of the embedded binary direction discrimination task showed signs of inter-item interactions with the item of the framing continuous direction report task beyond direct effects onto the percept of the embedded stimulus that happened prior to consolidation into working memory. To this end, we compared the shift of the mean PSE as a measure of a systematic bias and *SD* as a measure of precision for the presentation conditions and similarity steps. In line with a common workspace hypothesis we found clear evidence for interactions between the items within working memory, as shown by an increased repulsive bias as well as a similarity-tuned decrease of precision during concurrent memory retention.

Figure 1b (top) shows the mean PSEs of the embedded item in the perceptual and working memory condition, with positive values indicating that the motion direction stored for the framing task (first item) repelled the motion direction stored for the embedded task (second item). We calculated a repeated-measures ANOVA with the two within-subject factors Processing Level (perception vs. working memory) and similarity (30° vs. 60° vs. 90°). There was a significant main effect of inter-item similarity, *F*(1.35, 22.94) = 15.20, *p* < .001, η_p_^2^ = 0.47 (Greenhouse–Geisser corrected). The similarity tuning indicates an interaction between the items due to their common feature information—that is, motion direction. In addition, we found that the repulsive bias was significantly stronger in the memory condition as compared to the perceptual condition, *F*(1, 17) = 6.94, *p* = .017, η_p_^2^ = 0.29. This highlights that the repulsive interaction process continued after consolidation into working memory. There was no significant interaction between the factors, *F*(2, 34) = 0.24, *p* = .785, η_p_^2^ = 0.01. Together, these results revealed a similarity-tuned item interaction that already affected the perceptual representation and proceeded in an additive fashion during concurrent memory retention.

In addition to the interaction between the framing and the embedded items with regard to the repulsive bias, we also observed that the framing item affected the precision of the embedded item. However, the pattern of effects on precision differed from that of the repulsive bias. Figure 1b (bottom) shows the mean *SD* for perceptual and working memory representations for each similarity condition. Specifically, by calculating a repeated-measures ANOVA with the two within-subject factors Processing Level (perception vs. working memory) and inter-item similarity (30° vs. 60° vs. 90°) we found an overall larger *SD* in the working memory condition as compared to the perceptual condition (main effect of Processing Level), *F*(1, 17) = 6.70, *p* = .019, η_p_^2^ = 0.28, and an overall larger *SD* for similar than dissimilar items (a significant main effect of inter-item similarity), *F*(2, 34) = 4.62, *p* = .017, η_p_^2^ = 0.21. Importantly, in contrast to the repulsive bias we also found a significant interaction effect, *F*(2, 34) = 3.61, *p* = .038, η_p_^2^ = 0.18, showing that the precision of the embedded item was lower under the memory condition than under the perceptual condition for high inter-item similarity only. Post hoc paired *t* tests showed a significant difference for 30°, *t*(17) = 3.27, *p* = .005, Cohen’s *d* = 0.77, but no statistically meaningful difference for 60°, *t*(17) = 1.71, *p* = .106, Cohen’s *d* = 0.40, and for 90°, *t*(17) = 1.00, *p* = .331, Cohen’s *d* = 0.24.

### Discussion Experiment 1

Experiment 1 showed that inter-item competition affects memory representations even between items that are stored for distinct tasks that must be performed at distinct time points. We observed two types of item interactions. One of them affected the location of the direction representation in feature space (systematic bias), while the other interaction concerned the variability of the stimulus representation across trials (precision).

Regarding the systematic bias, we observed a repulsive shift of the item in the embedded task away from the item in the framing task. This repulsion effect showed the known similarity tuning, with an increase of magnitude with increasing similarity. Notably, the item in the embedded task was presumably already repelled on the perceptual level, as evident by the observed repulsion effect in the perceptual condition where the RDP and the reference point of the direction discrimination task were simultaneously present on the screen. In the working memory condition, this shift away from the memory item of the framing task increased during 1 s of concurrent maintenance, demonstrating that both items kept interacting on the memory level after perceptual encoding, even though the item of the embedded task was encoded last and probed first.

We also observed a similarity-tuned effect on the precision of the motion direction in the embedded task. The width of the psychometric function increased with increasing similarity in the memory condition. Notably, the precision of the perceptual representation of Sample 2 was unaffected by similarity. This shows that the precision decrease in working memory was not due to the temporal aspect of having a memory delay, as this would have led to an increase of the *SD* irrespective of the similarity between the memoranda. In contrast, this pattern demonstrates a specific effect of inter-item interaction that happened *exclusively* after stimulus encoding into working memory. Sample 1 of the framing task worsened the representation of Sample 2 in the embedded task only when both samples had similar motion direction *and* were concurrently maintained in memory.

Do our data present convincing evidence for a repulsion effect in the perceptual condition? Not necessarily. The present data could overestimate the repulsive effect on the percept. As the exposition duration was limited to 0.5 s, subjects might have experienced some trials where they were not able to identify the directional relation between Sample 2 and the reference point immediately and relied on a lasting working memory representation to finish decision-making instead. Thus, overall responses in the perceptual condition could be a composite of trials with responses based on undistorted perceptual representations and trials with responses based on distorted memory representations. However, if that were the case, the data would support the conclusion of repulsive interactions between memory items stored for distinct tasks even more strongly, since all the repulsive interaction would then have stemmed from competition on the memory level.

There are two limitations of Experiment 1 regarding the differences between the perception and working memory condition. First, both conditions were administered in a block design. Thus, participants were always fully aware of the type of the upcoming embedded task. Both conditions had slightly different demands with respect to the timing between encoding of the second stimulus, presence of the reference point, and decision-making in the direction discrimination task. This knowledge might have influenced encoding or maintenance strategies of the first stimulus, as well as the allocation of spatial attention during the processing of the second stimulus. It is reasonable to assume that spatial attention in the perception condition, where the reference was simultaneously on the screen with the RDP, was predominantly allocated towards the location of the reference point during stimulus processing, whereas no such a spatial anchor existed in the working memory condition during stimulus presentation. These differences might have differentially affected the representations of the stimuli and consequently the interaction profile. Second, due to the concurrent presence of reference and RDP in the perceptual condition, the processing of the motion direction might have qualitatively differed from the processing in the memory condition. Since the direction discrimination task required to judge the deviation of the motion direction with respect to the reference, participants might have relied on a reference-relative encoding strategy in the perceptual condition (i.e., towards which side does the motion direction deviate from the reference dot), rather than processing the motion direction in absolute terms (i.e., in what direction does the RDP move) and subsequently comparing it to the reference, which presumably was the order of processing in the memory condition. Different strategies in decision-making could of course elicit different interaction profiles. To confirm the results of Experiment 1 while avoiding its shortcomings, we conducted a second experiment.

## Experiment 2

In Experiment 2, we replaced the perception task with an iconic memory condition and shifted from a block design to a randomly interleaved presentation of conditions. Both changes led to the consequence that subjects could not tell what condition they were in until the offset of the second item. This ensured that stimulus processing was identical in both conditions until the second stimulus was perceptually processed. The replacement of the perceptual condition with an iconic memory condition avoided a simultaneous presentation of the reference probe and RDP that was a major difference between the perception and working memory conditions in Experiment 1. Second, by shifting from a block design to a randomly interleaved design with an unpredictable order of iconic memory and working memory trials, we avoided any condition-dependent stimulus processing of the items.

We chose to employ iconic memory as a substitute for the perceptual condition because iconic memory representations are thought to be close to sensory perceptual processing (Becker et al., 2000; Coltheart, 1980; DiLollo, 1977; Irwin & Yeomans, 1986; Lamme, 2010; Long, 1980; Sperling, 1960) and are formed prior to consolidated working memory representations (Awh et al., 2007; Lamme, 2004; Patterson et al., 2007). Thus, if the increased repulsion and decreased precision in the working memory condition of Experiment 1 were in fact based on interactions on the working memory level, iconic memory representations should produce a similar pattern of results as the perception task in Experiment 1.

### Methods

#### Participants

Twenty-four adults (14 females; age 19–39 years; *M* = 25.48, *SD* = 5.22) participated in the experiment after giving written informed consent. The study was approved by the local ethics committee. All participants reported normal or corrected-to-normal visual acuity. They were naïve to the purpose of the experiment and were either paid (€10/hr) or received course credit for their participation. Three subjects were excluded from data analysis. For two subjects, the curve-fitting procedure failed to estimate psychometric functions. Upon visual inspection, data of the two participants were consistent with a complete guessing response strategy. One subject had an outlier width of the psychometric function of >2.5 standard deviations of the mean. This left data from the remaining 21 participants (14 females; age 19–39 years; *M* = 25.10, *SD* = 4.74) for analysis. The sample size of experiment 2 followed the same rationale as in the first experiment.

#### Stimuli, apparatus, and data analysis

Stimuli, apparatus, and data analysis were identical to those in Experiment 1.

#### Procedure

**Fig. 2:**
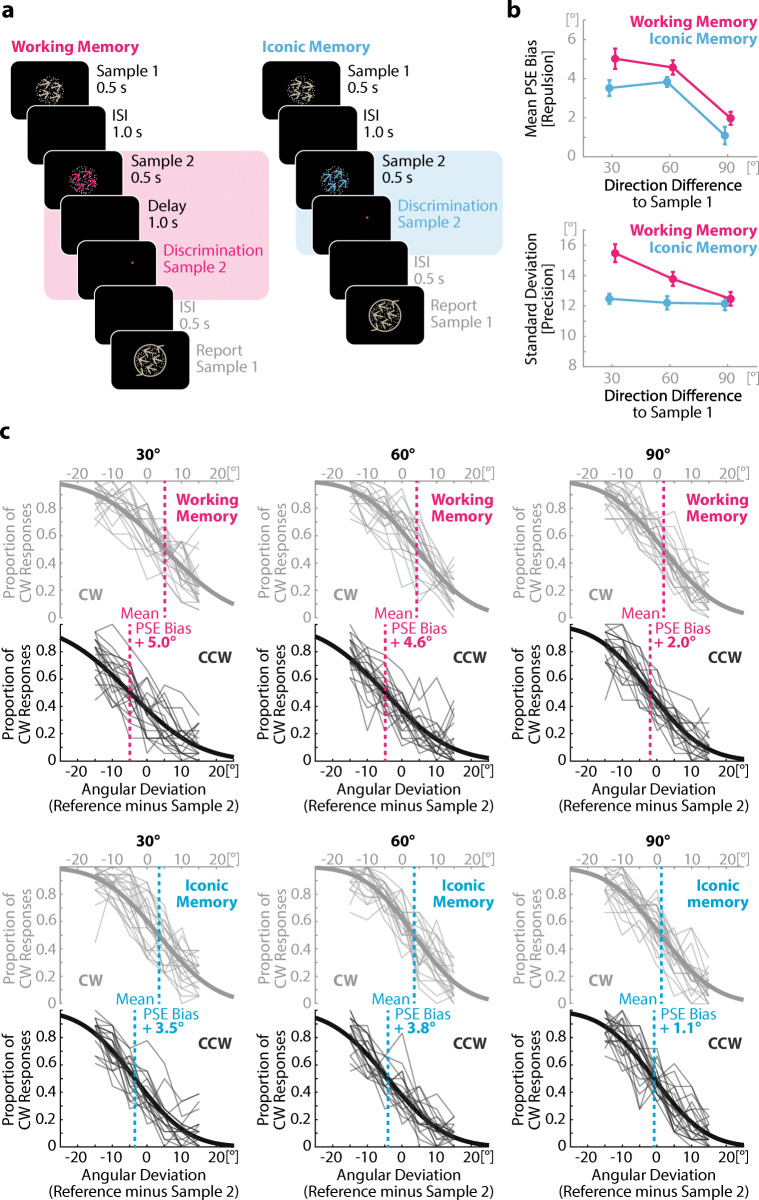
Design and results of Experiment 2. **a** Schematic depiction of an experimental trial of Experiment 2 in the working memory (left) and iconic memory (right) condition. In both conditions, subjects first memorized one motion direction of a random-dot-pattern (Sample 1; arrows only for illustration) for a continuous report at the end of the trial (framing task). For the embedded task, they memorized a second motion direction (Sample 2) for either a one-second delayed (working memory) or an immediate (iconic memory) binary discrimination. Note that the colors in the trial depiction are for illustrative purposes only. Both RDPs were equally white in the experiment. **b** The results of Experiment 2 replicated the results of Experiment 1 for both repulsive bias (top) and precision (bottom). Specifically, the memory item in the embedded task was repulsed by the memory item of the framing task in both conditions, as indicated by a shift of the PSE away from the motion direction. This repulsion effect was stronger for higher similarity between both samples and for the working memory as compared with the iconic memory condition. The precision of Sample 2 was lower (higher *SD*) in the working memory than iconic memory condition. This effect, however, was strongly modulated by the motion direction similarity between both samples. Error bars represent within-subject standard errors (Morey, 2008). **c **Detailed visualization of the repulsive bias including individual subjects’ data. Mean PSE bias was calculated as half the distance between the PSE of trials in which Sample 2 was oriented clockwise relative to Sample 1 (CW) and the PSE of trials in which Sample 2 was oriented counterclockwise relative to Sample 1 (CCW). The figures in **c** show the mean performance on the direction discrimination task at the level of individual subjects (thin lines) for CW and CCW trials, respectively. Superimposed is the mean psychometric function (thick line). The dashed line shows the point of subjective equality (PSE) per condition. For CW trials, a shift of the psychometric function to the right indicates repulsion of Sample 2 from Sample 1; for CCW trials, a shift to the left indicates repulsion. (Color figure online)

Figure 2a depicts the trial structure. The procedure was identical to Experiment 1, except for the following changes: The perception condition of Experiment 1 was replaced by an iconic memory condition. In the iconic memory condition, the reference point (red dot) appeared immediately after the offset of the second stimulus and remained visible until response. The experiment consisted of 1,512 experimental trials, 756 trials per condition (iconic memory, working memory) comprising 252 repetitions per inter-item similarity step (30°, 60°, 90°), with 36 repetitions per reference probe deviation each. Participants performed 30 practice trials at the beginning of each session that were excluded from data analysis. Conditions were presented randomly interleaved, without information about the upcoming condition, to ensure that the cognitive processes were identical for both conditions until offset of the second item. The experiment lasted about 2 hours per session and comprised two sessions on different days.

### Results

Experiment 2 tested whether we could replicate results of Experiment 1 when the reference point appeared immediately after (iconic memory) instead of during (perception) the offset of the second sample and when conditions were mixed instead of blocked during the experiment. We found again clear evidence for interactions between items within working memory, as shown by an increased shift of the PSE as well as a similarity-tuned decrease of precision during concurrent memory retention.

Figure 2b (top) shows the mean PSEs of Sample 2 in the working memory and iconic memory conditions, with positive values indicating that the motion direction stored for the framing task (Sample 1) repelled the motion direction stored for the embedded task (Sample 2). The repulsion was significantly stronger in the working memory than in the iconic memory condition (main effect of Processing Level), *F*(1, 20) = 12.72, *p* = .002, η_p_^2^ = 0.39, and stronger for similar than dissimilar items (main effect of inter-item similarity), *F*(1.36, 27.12) = 19.53, *p* < .001, η_p_^2^ = 0.49 (Greenhouse–Geisser corrected). However, there was no interaction between both factors, *F*(2, 40) = 0.68, *p* = .510, η_p_^2^ = 0.03. Figure 2b (bottom) depicts the mean *SD* in the working memory and iconic memory conditions for each similarity condition. Again, we found an increased *SD* in Sample 2 in the working memory compared with the iconic memory condition (main effect of Processing Level), *F*(1, 20) = 14.25, *p* = .001, η_p_^2^ = 0.42. We also replicated the increased *SD* for more similar combinations of Samples 1 and 2 (main effect of inter-item similarity), *F*(2, 40) = 6.02, *p* = .005, η_p_^2^ = 0.23. As in Experiment 1, the precision of Sample 2 was lower under the working memory condition than under the iconic memory condition for high inter-item similarity only, interaction, *F*(2, 40) = 4.42, *p* = .018, η_p_^2^ = 0.18. Post hoc paired *t* tests showed significant differences for 30°, *t*(20) = 4.16, *p* < .001, Cohen’s *d* = 0.91, and 60°, *t*(20) = 2.39, *p* = .027, Cohen’s *d* = 0.52, but not for 90°, *t*(20) = 0.49, *p* = .626, Cohen’s *d* = 0.11.

### Discussion Experiment 2

In Experiment 2, we aimed to replicate the results of the first experiment, while eliminating confounding factors. Experiment 2 replicated the results of the first experiment to a striking degree of similarity. We observed clear evidence for competition between consolidated working memory representations. This competition was, again, signified by a stronger repulsion bias and a reduced precision of a subsequently integrated working memory representation, compared to a subsequently processed iconic memory representation. As in Experiment 1, precision was only affected by item similarity in the working memory condition, indicating a similarity-tuned interference effect that originates exclusively on the working memory level. The repulsive shift of the second item showed again a similarity tuning in both conditions but with a generally stronger repulsion after concurrent maintenance in working memory. Taken together, Experiment 2 provided further evidence that task-separated item representations keep interacting after consolidation into working memory.

## General discussion

Common models of working memory conceptualize it as a shared space in which representations are placed, as equals, until they are needed for recall. This concept explains capacity limitations and interference effects within working memory, as there is only limited space available along a common organizational principle, usually feature dimensions. Thus, similar representations being close or overlapping in feature space, rely on the same resources for representation and consequently interfere with one another (e.g., Compte et al., 2000; Franconeri et al., 2013; Johnson et al., 2008; Lin & Luck, 2009; Oberauer & Lin, 2017; Schneegans & Bays, 2017; Swan & Wyble, 2014). This concept fits well with the experimental designs normally employed to study visual working memory in which all memory items are task equivalent as they all need to be maintained concurrently as the potential target for the same report task until the cue or probe appears. Thus, all items are stored as candidates for the same purpose at the same time with no indications to organize them along anticipated behavioral demands.

In contrast, recent action accounts of working memory have emphasized the behavioral application specificity or goal-directedness of working memory representations. In this view, working memory representations are not conceived of as a sensory preserve of past stimulation but as an anticipation of future demands. Working memory representations thus do not (exclusively) represent the way an object appeared, but the way it is needed to solve the specific future task for which the information was sought and stored in the first place (e.g., Heuer et al., 2020; Myers et al., 2017; Nobre & Stokes, 2019; van Ede, 2020). Evidence for this account has been presented in numerous studies that show how future behavioral demands alter stimulus processing during encoding and maintenance according to when (Christophel et al., 2018; de Vries et al., 2020; LaRocque et al., 2017; Lewis-Peacock et al., 2012; Olivers & Roelfsema, 2020; Peters et al., 2018) and how (Heuer et al., 2020; Monaco et al., 2018; Muhle-Karbe et al., 2017; Nobre & Stokes, 2019; Warden & Miller, 2010) stimulus information will be needed.

In contrast to the common workspace view of working memory, the action perspective suggests that memory representations are separated along anticipated temporal and behavioral coordinates. Here, we tested two measures of inter-item competition (i.e., representational bias and similarity-induced decrease of precision) under ideal circumstances for item separation by task (continuous report versus binary discrimination) and timing demands (encoded first and probed second versus encoded second and probed first) and carefully controlled for effects that occur already prior to working memory consolidation, to uncover unique interference effects during concurrent working memory maintenance.

Each item was encoded separately at a distinct point in time, was task-relevant for a unique behavioral task, and was due for action at a distinct predetermined timepoint that remained constant across all trials. Despite the high degree of contextual distinctiveness, we observed clear interference effects at the working memory level. During concurrent maintenance of the items there was an overall increased repulsive bias of the direction representation and a memory-specific deterioration of item precision when both memory items were similar to each other. The results clearly suggest that even separately encoded items, that are stored for distinct tasks and are due at distinct predetermined points in time, reside in a common workspace, as evidenced by the observed item interactions that occurred specifically on the memory level.

One limitation in the interpretation of our results is that we cannot exclude the possibility that the stimuli have been processed and memorized in relative terms. In fact, one could, for example, code the direction of the second stimulus as a signed angular deviation from the first, rather than storing the stimuli merely as absolute directions. In that case, both items would be related to one another simply due to the coding strategy, which could have ongoing effects on the memory representations. Even though it is not immediately apparent how such a coding strategy could be beneficial to solving the direction discrimination task, especially given its temporal priority in task order, such an encoding mode could be obligatory and out of reach of strategic cognitive processing. But even then, our central conclusion would hold that the items were stored together even when belonging to different task sets. Furthermore, both tasks of our paradigm shared many common features. While the task-related distinctiveness of the items in our experiments was certainly stronger than in common working memory studies where items are stored concurrently for the same task, they were still highly similar. Future research should focus on the generalizability of our findings to different degrees of similarity of items and encoding contexts to get a better picture of the boundary conditions of the interactive patterns we observed in our study.

With respect to the action perspective of working memory, we would like to stress that our results do not challenge this account. First of all, memory content might well be represented concurrently in several formats (Boettcher et al., 2021; Yan et al., 2021). Hence, representations that are optimized for the specific upcoming task demands might coexist and interact with other (e.g., more sensory-like) representations. Our finding of inter-item competition does not allow the conclusion that no action-specific representations were formed and maintained. Rather, it tells us that at least one format of memory representations existed in which the items stayed in contact during the common maintenance phase, despite both items belonging to different task sets. Second, while the interacting stimuli in our experiments were clearly distinguishable with respect to temporal, task, and response characteristics, they did not allow for a transformation into unambiguous and differential action codes prior to maintenance, as the continuous direction report task and the binary direction discrimination task both required the respective direction information of the stimuli at the time of the response. The sensory information of the stimuli (or its directional derivatives) was potentially maintained until the respective response was due. Thus, even when belonging to different task sets, stimulus information was not kept strictly separated. This suggests that different task sets do not necessarily open up distinct representational spaces but that upcoming information is initially integrated into a common store. Task-specific item selection and response preparation might then be achieved in a second step via binding of relevant temporal and action-related task features to the sensory memory information. This interpretation converges with two recent studies that showed evidence of a dual coding strategy of action-oriented and sensory information in visual working memory tasks. Dual coding seems to be maintained even when the presented visual information can be unambiguously transformed into an action code prior to the delay period and thus becomes, in principle, irrelevant for task completion (Henderson et al., 2022). Both types of representations seem even to be formed and maintained in expectation of an intervening task (Boettcher et al., 2021). The action dimension of a stimulus within a specific task might thus not be strictly separated from the initially encoded sensory information but remains in a bound relationship (Olivers & Roelfsema, 2020; van Ede, 2020). And third, a recent study by Gresch et al. (2022) showed that the response latency in an embedded perceptual discrimination task (similar to the perceptual task in our Experiment 1) was modulated by the anticipated temporal onset of upcoming retrieval demands for a framing working memory task (similar to our framing task) even though both tasks were independent in task and response format. This suggests that the cognitive associations between intertwined tasks go beyond questions of (in-)dependent stimulus representations. Temporally nested tasks seem to get cognitively related on several levels that comprise, among others, strategic encoding preparation prior to the onset of intervening stimuli (Gresch et al., 2021), stimulus processing during perceptual encoding (Kang et al., 2011; Scocchia et al., 2013), as well as response preparation and execution (Gresch et al., 2022).

Our study also enriches other debates in working memory research. A recent study by Scotti et al. (2021) showed that simultaneously encoded items that are maintained concurrently as potential targets for the same recall task, repel each other uniquely during working memory maintenance, as the repulsion effect was only observable after a prolonged concurrent retention period. Our study adds to this finding by showing that feature repulsion during memory maintenance does not depend on the common encoding context of simultaneous presentation. Our observation of delay-period specific repulsion even after *sequential* encoding of memory items suggests that the mechanisms that are responsible for repulsive interactions during concurrent memory maintenance are independent of the temporal relationship during encoding (see Czoschke et al., 2020, for a more nuanced discussion of this topic). Furthermore, the similarity tuning of the precision loss in our study was a specific interference-effect of memory maintenance. Item similarity did not affect the precision of perceptual or iconic memory representations, even though similarity was correlated with the magnitude of the repulsive shift. This observation suggests that independent interference mechanisms act on the repulsive bias on the one hand and precision of representations on the other hand. A representational bias, as seen in the perceptual and iconic memory conditions, can occur without a corresponding decrease in precision. Such independence of bias and precision has previously been shown for random drifts of working memory representations (i.e., nonrelated to other items) during delay periods (Panichello et al., 2019). Whether the same independence also holds for the increased item-induced repulsive drifts within working memory needs to be addressed in future research. Our results are also not compatible with the proposed benefit of high inter-item similarity for item precision due to a sharpening of the demarcation of item borders on the facing side of concurrent representations (Lin & Luck, 2009) or due to decoding principles of multiple memory signals in a shared binding space (Oberauer & Lin, 2017). Finally, the fact that the detrimental effect of similarity on precision was specific to the memory level, but did not appear prior to consolidation, further indicates that interaction processes within working memory are not a mere continuation of perceptual interaction mechanisms that transcend onto the memory level. In contrast, unique interference mechanisms seem to exist within working memory. Recent research has presented a similar conclusion in the other direction, showing that interference effects between visual objects on the perceptual level are not elicited within working memory, opposing a central prediction following from the sensory recruitment hypothesis of working memory (Bloem et al., 2018; Czoschke et al., 2020; Harrison & Bays, 2018). Here, we add to the evidence for independent interference mechanisms in perception and working memory: Unique inter-item interference mechanisms, which led to a deterioration of precision in our study, appear to be elicited between concurrently maintained representations on the working memory level.

In summary, our results show that items that are stored in working memory interact with each other even if they are encoded and retained for different actions. This finding indicates a common workspace in working memory where items are stored and integrated by default. Moreover, interactions between items in working memory can lead to systematic biases and precision loss of item representations. These detrimental effects occur independently of each other: The systematic bias might originate from sensory interactions that continue in working memory, whereas the observed precision deterioration points toward an interference mechanism that operates exclusively in working memory.
